# Correlates of retinopathy in persons living with type 2 diabetes at a clinic in Zambia

**DOI:** 10.11604/pamj.2024.49.83.43657

**Published:** 2024-11-21

**Authors:** Joyce Kukeng'a Mwangu, Lukundo Siame, Memory Ngosa, Warren Chanda, Benson Malambo Hamooya, Bwalya Bupe Bwalya, Sepiso Kenias Masenga

**Affiliations:** 1 HAND Research Group, School of Medicine and Health Sciences, Mulungushi University, Livingstone, Zambia,; 2 Department of Economics, School of Social Science, Mulungushi University, Kabwe, Zambia,; 3 Demography and Population Studies Programme, Schools of Public Health and Social Sciences, University of the Witwatersrand, Johannesburg, South Africa

**Keywords:** Diabetic retinopathy, retinopathy, type 2 diabetes mellitus, Zambia

## Abstract

**Introduction:**

diabetes mellitus presents a significant public health challenge globally, with its prevalence projected to rise, particularly in developing countries. Diabetic retinopathy is a common complication of diabetes and a leading cause of blindness among adults. Despite its significance, research on diabetic retinopathy in sub-Saharan Africa, including Zambia, remains limited. This study aimed to determine the prevalence of diabetic retinopathy and identify associated factors among individuals with type 2 diabetes mellitus (T2DM) attending Mahatma Gandhi Clinic in the Livingstone District of the Southern Province of Zambia.

**Methods:**

this was a cross-sectional study, involving 48 participants aged 18 years and above, living with T2DM, and enrolled at Mahatma Gandhi Clinic in Livingstone District of Southern Province, Zambia. The primary outcome was diabetic retinopathy, which was classified using the international classification of diabetic retinopathy. Both univariate and bivariate analysis were used to estimate the prevalence of diabetic retinopathy, whereas, logistic regression was used to determine the bio-demographic, social, and clinical variables associated with diabetic retinopathy.

**Results:**

the majority were females (n=37, 77.1%). The median age was 54 years (interquartile range (IQR) 48, 65). The mean Body Mass Index (BMI) was 27.4 (standard deviation (SD), ±6.0). The median duration of living with T2DM was 60 months (IQR, 36,132), with a mean pulse pressure of 9.6 ± 2.8 mmHg and mean glycosylated hemoglobin (HBA1C) of 9.58 ± 2.77%. Fifty percent of the participants had visual problems and hypertension (n=24 each, 50%,). Most participants reported not exercising for at least 30 minutes (n=37, 77.1 %,) and had uncontrolled sugar levels (n=32, 66.7%). Of the 48 participants, 12 (25%) had diabetic retinopathy. Longer duration of T2DM was the only factor significantly associated with diabetic retinopathy on multivariable analysis (AOR: 1.01; 95%Cl 1.00, 1.03, p<0.038).

**Conclusion:**

this study highlights a high prevalence (25%) of diabetic retinopathy among people living with type 2 diabetes mellitus (T2DM) being managed at Mahatma Gandhi Clinic in the Livingstone District of the Southern Province of Zambia. Longer duration of living with T2DM was positively associated with diabetic retinopathy. There is a need to provide routine eye examinations in this population and increase knowledge among clients living with T2DM to reduce the burden of diabetic retinopathy in our setting.

## Introduction

Diabetes mellitus is a public health challenge [1]. The prevalence of diabetes globally is expected to increase to about 439 million adults by 2030, affecting mostly developing countries [2]. The surge in diseases is correlated with a rise in obesity in developing countries [3]. Diabetes leads to long-term complications that include retinopathy, neuropathy, and nephropathy [4]. Diabetic retinopathy is a prevalent microvascular pathology characterized by changes in the retina, and it ranks as a leading cause of blindness within adult working populations [1,5]. Zambia, like other developing countries, is facing a double burden of non-communicable diseases and communicable diseases, with diabetes on the rise due to an increased prevalence of obesity, reaching approximately 19% [6-8]. The estimated prevalence of diabetes among the adult population is about 3.5%, with diabetic retinopathy affecting approximately 52% [6]. However, despite the increasing burden of type 2 diabetes mellitus (T2DM) and diabetic retinopathy, a large proportion are not receiving treatment and diagnostic challenges remain in most parts of Zambia [7].

While numerous studies have investigated retinopathy in people living with diabetes, there remains a scarcity of research in sub-Saharan Africa (SSA) and specifically in Zambia that addresses the factors associated with diabetic retinopathy among patients with T2DM. Therefore, the objective of this study was to determine the prevalence of diabetic retinopathy and identify the associated factors among individuals living with T2DM at Mahatma Gandhi Clinic in Livingstone District, of the Southern Province in Zambia.

## Methods

**Study design and setting:** this was a cross-sectional study that was conducted at Mahatma Gandhi Clinic. One of the largest clinics in Livingstone District, Southern Province, Zambia.

**Sample size, eligibility, and sampling:** the number of people living with T2DM aged above 18 years enrolled in the clinic was about 50. We conducted a census of all participants and invited all people with diabetes mellitus to participate after signing a written consent. A total of 48 consented to participate in the study.

**Variables:** the primary outcome variable was diabetic retinopathy (binary). Diabetic retinopathy was diagnosed with a visual acuity test followed by a fundoscopy examination performed by an ophthalmologist. Diabetic retinopathy was classified using the International Classification of Diabetic Retinopathy (ICDR) [9]. Independent variables considered in the study included bio-demographic and social factors (age, gender, marital status, educational level, duration of DM, employment, exercise, and alcohol), and clinical parameters (hypertension, glucose levels, pulse pressure, visual problem, and glycosylated hemoglobin).

**Data collection:** data for the study were collected between July and December 2023 using a structured questionnaire, supplemented by parameters abstracted from patients' files. Two (2) research assistants were responsible for data collection, and the senior supervisor ensured data accuracy at the end of each day.

**Data analysis:** the data collected were entered into Microsoft Excel 2013, where it underwent cleaning and coding for analysis. Analysis was conducted using GraphPad Prism version 7 and SPSS version 13. Descriptive statistics, including frequencies, percentages, means, medians, and standard deviations or interquartile ranges, were utilized to summarize the results. To assess the normality of the data, histograms were initially employed, followed by the Shapiro-Wilk test. For categorical variables, the Chi-square test was applied. To determine statistical differences for continuous variables, the t-test was utilized for normally distributed variables, whereas the Mann-Whitney U-test was employed for non-normally distributed variables. A binary logistic regression model was subsequently employed to establish the relationship between outcome variables and independence. Only variables that were statistically significant and had clinical relevance were included in univariable and multivariable analysis.

**Ethical consideration:** ethical approval was obtained from Mulungushi University School of Medicine and Health Sciences Research Ethics Committee (MU-SoMHS-REC: Reference number SMHS-MU2-2023-91) on 15^th^ June 2023 and permission was granted by Mahatma Ghandi clinic management before commencement of data collection. Written consent was obtained from the participants and no identifying information was obtained from the participants such that no participant was identified during or after the data collection or during analysis. Strengthening the report of observational studies in epidemiology (strobe) for reporting observational study was used to guide our reporting.

## Results

**Descriptive characteristics and relationship between diabetic retinopathy and sociodemographic and clinical characteristics:** the study comprised 48 participants, of whom 77.1% (n=37) were females, and the median age was 54 years (interquartile range, IQR 48, 65) (Table 1). The mean body mass index was 27.4±6.0 kg/m^2^. A total of 29 (60.4%) participants were married, and the majority of the participants had a secondary school education level (n=18, 37.5%). Thirty-nine (39 (81.3%)) participants were unemployed. The majority had a history of having consumed alcohol (n=43, 89.6%). The proportion of participants with or without visual problems was comparable (n=24, 50%), like those with and without hypertension (n=24, 50%). The majority of participants reported not exercising for at least 30 min (n=37, 77.1%). Most participants had uncontrolled fasting blood glucose (n=32, 66.7). The median duration of T2DM among participants was 60 (36,132) months. The mean pulse pressure was 9.6±2.8 mmHg, and the mean glycosylated hemoglobin (HBA1C) was 9.58±2.77%. The prevalence of diabetic retinopathy was 25% (n=12). Participants with diabetic retinopathy had T2DM longer than those without retinopathy (131 vs. 77 months, p<0.016). Participants with diabetic retinopathy did not exercise for at least 30 minutes compared to those who were exercising (12 vs. 0, p<0.029) and had a higher pulse pressure compared to those without diabetic retinopathy (64.4 vs. 51 mmHg, p<0.003).

**Table 1:** descriptive characteristics and presence of diabetic retinopathy

Variable	Median, (IQR) OR Frequency (%)	Presence of Retinopathy		P-value
		Yes (n=12, 25%)	No (n=36, 75%)	
**Age (years)**	54(49, 65)	60(48, 70)	51(48, 63.5)	0.492
**Sex**				
Male	11(22.92)	2(16.67)	9(25.00)	0.552
Female	37(77.08)	10(83.33)	27(75.00)	
**BMI, mean (±SD)**	27.4 (6.0)	26.2(5.7)	27.9(61.7)	0.294
**Marital status**				
Single	3(6.25)	0(00)	3(8.33)	0.302
Married	29(60.42)	9(75.00)	20(55.56)	
Divorced	5(10.42)	2(16.67)	3(8.33)	
Widowed	11(22.92)	1(8.33)	10(27.78)	
**Duration of DM, months**	60(36,132)	131(76.84)	77(61.65)	0.016
**Level of education**				
No education	16(33.33)	3(25.00)	13(36.11)	0.715
Primary	1(2.08)	0(2.08)	1(2.78)	
Secondary	18(37.50)	18(37.50)	12(33.33)	
Tertiary	13(27.08)	13(27.08)	10(27.78)	
**Currently employed**				
No	39(81.25)	10(83.33)	29(80.56)	0.831
Yes	9(18.75)	2(16.67)	7(19.44)	
**Visual problems**				
No	24(50.0)	2(33.33)	20(55.56)	0.182
Yes	24(50.0)	8(66.67)	16(44.44)	
**Hypertensive retinopathy**				
No	13(68.4)	0(00)	13(100)	0.316
Yes	6(31.6)	1(16.7)	5(83.3)	
**Hypertensive**				
No	24(50.0)	6(50.00)	18(50.00)	0.831
Yes	24(50.0)	6(50.00)	18(50.00)	
**Exercise for 30 minutes**				
No	37(77.08)	12(100)	25(69.44)	0.029
Yes	11(22.92)	0(00)	11(30.56)	
**Consume alcohol**				
No	43(89.56)	11(89.67)	23(63.89)	0.862
Yes	5(10.42)	1(8.33)	13(36.11)	
**Controlled sugar levels**				
No	32(66,7)	7(58.33)	25(69.44)	0.480
Yes	16(33.33)	5(41.67)	11(30.56)	
**HBA1C, mean (SD)**	9.6(2.77)	9.8(3.06)	9.5(2.72)	0.895
**Pulse pressure, mean (SD)**	64(18.41)	64(18.41)	51(17.6)	0.003

DM: diabetes mellitus; HBA1C: glycated hemoglobin; SD: standard deviation

**Figure 1:**
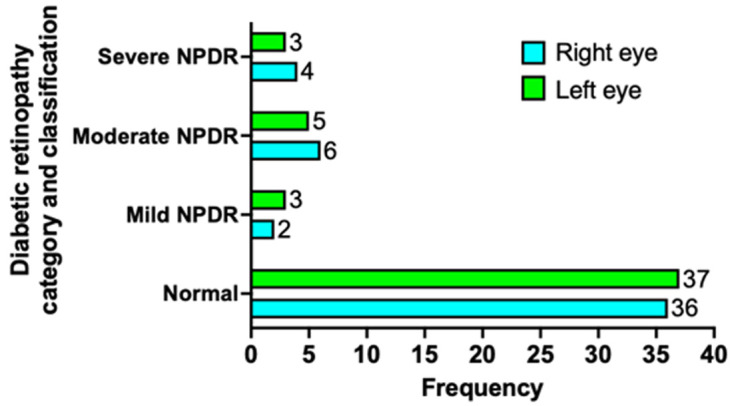
retinopathy classification by eye; NPDR: non-proliferative diabetic retinopathy

**Retinopathy classification:** 75% (n=36) and 77% (n=37) of patients had no abnormalities in the right and left eye, respectively (Figure 1). Those with severe non-proliferative diabetic retinopathy (NPDR) on the right and left eye were 8.3% (n=4) and 6.23% (n=3), respectively. About 12.5% (n=6) and 10.4% (n=5) had moderate NPDR, respectively.

**Univariable and multivariable logistic regression of factors associated with diabetes mellitus:** at univariable analysis, for every unit increase in the duration of type 2 diabetes mellitus (T2DM), the odds of having diabetic retinopathy increased by 1% (odds ratio (OR): 1.01, 95% confidence interval (Cl), 1.00, 1.03, P<0.038) (Table 2). For every unit increase in pulse pressure, the odds of having diabetic retinopathy increased by approximately 3% (OR: 1.03; 95% CI: 1.00, 1.07). Only duration of diabetes was significantly associated with diabetic retinopathy at multivariable analysis where the odds of having diabetic retinopathy increased by 1% for every unit increase in the duration of T2DM (adjusted odd ratio (AOR): 1.01; 95%Cl 1.00, 1.03, p<0.038).

**Table 2:** univariable and multivariable logistic regression of factors associated with diabetic retinopathy

	Univariable analysis		Multivariable analysis	
Variables	OR (95%CI)	P-value	AOR (95%, Cl)	P-value
**Age**	1.02 (0.96-1.09)	0.375	1.00 (0.92-1.09)	0.905
**Sex**				
Male	1		1	
female	1.85 (0.34-9.96)	0.473	2.52 (0.20-31.78)	0.474
**Duration of T2DM, months**	1.01 (1.00-1.02)	**< 0.028**	1.01 (1,00-1.03)	**0.038**
**Hypertension**				
Normotensive	1		1	
Hypertension	1.00 (0.27-3.69)	1.00	0.19 (0.02-1.62)	0.130
**HBA1C**	1.03 (0.81-1.31)	0.762	1.10 (0.81-1.49)	0.511
**Pulse pressure**	1.03 (1.00-1.07)	**< 0.039**	1.05 (0.99-1.11)	0.054

T2DM: type 2 diabetes mellitus; HBA1C: glycated hemoglobin; SD: standard deviation

## Discussion

This study aimed to determine the prevalence and predisposing factors associated with retinopathy in persons living with T2DM at Mahatma Gandhi Clinic. The prevalence of diabetic retinopathy was 25%, with 8.3% on the right eye and 6.23% on the left eye having severe non-proliferative diabetic retinopathy (NPDR), and the only factor positively associated with diabetic retinopathy was duration of diabetes.

The current study found a 25% prevalence of diabetic retinopathy which closely matches the global estimate of 22.27%, as reported by Teo *et al*. (2021) [8]. This prevalence is higher than that of Europe at 20.6% and Southeast Asia at 12.5%, but lower than that of Africa at 33%, as reported by Thomas *et al*. (2019) [10]. Similar rates have been reported in Arab countries (22.5%) and Zimbabwe (28.4%) a country in the same region as Zambia [8,11,12]. While few studies have been conducted in Zambia on the prevalence of diabetic retinopathy, one study found a prevalence of 52% and another study in Zambia found a prevalence of 47.4 % slightly twofold higher than what we have reported in our study [6]. The primary factors contributing to the high prevalence in our environment are the limited availability of diabetes services and the fact that the majority of the participants were unable to purchase high-priced diabetic supplies, such as oral anti-diabetic medication and insulin, testing strips and glucose chips for frequent monitoring and thus a majority of our patients mostly depend on government supplies which are mostly rationed and shortages are common [13-16]. Moreover, many patients face challenges with proper insulin storage, as refrigeration is often unavailable, and if available, frequent power outages prevent proper storage, this results in challenges in control of hyperglycemia which in turn leads to diabetic retinopathy in our setting [17].

Secondly, many individuals in our area lack knowledge about diabetes-related symptoms and are unaware of the diagnosis; among those who are aware, the majority are not on treatment, and those who are on treatment have poor glycemic control and failure to understand and comply with treatment results in adverse outcomes [7]. This knowledge deficit, coupled with prevalent traditional beliefs that question the effectiveness of Western medicine or where patients simultaneously seek traditional and Western medicine, is common in our setting and is the case in sub-Saharan Africa and often leads to reliance on ineffective treatments and delays in seeking proper medical care. Moreover, inconsistent care-seeking behaviors and frequent switching between healthcare providers further undermine the continuity and effectiveness of diabetes management, potentially worsening patient outcomes like diabetes retinopathy [18].

In our study, the only factor associated with the development of diabetic retinopathy was the duration of diabetes. This factor has consistently been linked to the development of diabetic retinopathy, especially if glucose control is poor, like in our case [19,20]. The polyol pathway plays a crucial role in the development of diabetic retinopathy, a common microvascular complication of diabetes [21]. In this pathway, glucose is converted to sorbitol by aldose reductase, leading to sorbitol accumulation in retinal cells [22]. This accumulation causes osmotic and oxidative stress, resulting in cell damage and apoptosis. The duration of diabetes of more than 5 years and chronic hyperglycemia are key associated factors for diabetic retinopathy as was the case in this present study, as prolonged exposure to high glucose levels accelerates the polyol pathway [23]. The polyol pathway interacts with other pathogenic mechanisms, including advanced glycation end-product formation, protein kinase C activation, and inflammation, contributing to the complex pathophysiology of diabetic retinopathy [24]. Given the high prevalence of diabetic retinopathy, there is a need to set up diabetes clinics with qualified personnel in our setting, where routine eye clinics are done to prevent blindness and other diabetes complications.

Although we only reported one significant factor associated with diabetic retinopathy, several studies have demonstrated that factors associated with diabetic retinopathy include sex, age, diabetes duration, blood pressure, glycemic control, educational level, and hypertension [25,26]. These factors were not found to be significantly associated with our study. One possible reason could be the differences in methodology and sample size. Another potential reason could be regional differences in healthcare access or the socioeconomic status of our population, which may overshadow the effects of these traditional risk factors.

The study provided insight into the prevalence of diabetic retinopathy and its associated factors among people living with type 2 diabetes. However, the limitations of our study include a small sample size and cross-sectional design, thus, these results cannot be generalized nor causality ascertained. Therefore, further studies are necessary with a larger sample size, which should include more independent variables (treatment modality, number of visits, etc.).

## Conclusion

This study established a baseline prevalence of diabetic retinopathy in patients with T2DM receiving care at our health facility which is above the global prevalence with long duration of diabetes being positively associated. Thus, there is a need to enhance and strengthen awareness among people living with diabetes and conduct regular eye examinations among this cohort in our setting in order to prevent blindness.

### What is known about this topic

- The burden of diabetic retinopathy is known to be high in sub-Saharan Africa;
- The burden of diabetic retinopathy in Zambia is thought to be high;
- Challenges relating to the proper management of diabetes in our setting are well known.

### What this study adds

- This study provides local insight into the burden of diabetic retinopathy;
- Key local factors associated with diabetes have been identified;
- Our study contributes to the growing knowledge around diabetic retinopathy and serves as a basis for further research.
